# Shared and divergent principles of synaptic transmission between cortical excitatory neurons in rodent and human brain

**DOI:** 10.3389/fnsyn.2023.1274383

**Published:** 2023-09-05

**Authors:** Christiaan P. J. de Kock, Dirk Feldmeyer

**Affiliations:** ^1^Center for Neurogenomics and Cognitive Research, Vrije Universiteit Amsterdam, Amsterdam, Netherlands; ^2^Research Center Juelich, Institute of Neuroscience and Medicine, Jülich, Germany; ^3^Department of Psychiatry, Psychotherapy, and Psychosomatics, RWTH Aachen University Hospital, Aachen, Germany; ^4^Jülich-Aachen Research Alliance, Translational Brain Medicine (JARA Brain), Aachen, Germany

**Keywords:** excitatory neurotransmission, rodent, primate, human, synapse, EPSP, neocortex

## Abstract

Information transfer between principal neurons in neocortex occurs through (glutamatergic) synaptic transmission. In this focussed review, we provide a detailed overview on the strength of synaptic neurotransmission between pairs of excitatory neurons in human and laboratory animals with a specific focus on data obtained using patch clamp electrophysiology. We reach two major conclusions: (1) the synaptic strength, measured as unitary excitatory postsynaptic potential (or uEPSP), is remarkably consistent across species, cortical regions, layers and/or cell-types (median 0.5 mV, interquartile range 0.4–1.0 mV) with most variability associated with the cell-type specific connection studied (min 0.1–max 1.4 mV), (2) synaptic function cannot be generalized across human and rodent, which we exemplify by discussing the differences in anatomical and functional properties of pyramidal-to-pyramidal connections within human and rodent cortical layers 2 and 3. With only a handful of studies available on synaptic transmission in human, it is obvious that much remains unknown to date. Uncovering the shared and divergent principles of synaptic transmission across species however, will almost certainly be a pivotal step toward understanding human cognitive ability and brain function in health and disease.

Excitatory (glutamatergic) synaptic transmission is the primary mode of communication between principal neurons in the neocortex and allows information transfer between synaptically connected neurons. Local inhibitory (GABAergic) synapses modulate (i.e., reduce or disinhibit) the activity of neighboring principal neurons but do not directly contribute to information transfer, at least in the adult (Rheims et al., 2008; Kirmse et al., 2015). The basic blueprint of the synapse typically includes the neurotransmitter release machinery of the presynaptic axon and the associated receptor complex on the postsynaptic dendrite. However, this simplified scheme is easily extended to a spectrum of synapse types, including (i) axo-axonic synapses (Somogyi, 1977; Gonchar et al., 2002; Schneider-Mizell et al., 2021), (ii) axo-somatic synapses (Borst and Soria van Hoeve, 2012; Kubota et al., 2016), (iii) dendro-dendritic synapses [(Woolf et al., 1991; Aghvami et al., 2022)], or (iii) the tripartite synapse with juxta-posed astrocytes [(Araque et al., 1999; Liu et al., 2023)]. Furthermore, a single postsynaptic spine can be occupied by multiple presynaptic terminals and contact points are found between axonal bouton and postsynaptic spine neck or dendritic shaft (Cano-Astorga et al., 2021). Because the synapse is the fundamental locus for communication between neurons, it is not surprising that synaptic dysfunction leads to a plethora of brain disorders.

Structural features of individual synapses are studied at maximal spatial resolution using electron microscopy (EM), which can also provide information on the parent neuronal cell type and the local microcircuit (Kasthuri et al., 2015; Schmidt et al., 2017; Loomba et al., 2022). EM typically provides quantitative insight into absolute synapse number or synapse density, which in turn are specific to the brain region or sub-region (e.g., cortical layer) under investigation (DeFelipe et al., 2002). Single dendrite or single spine analysis using EM further uncovered branch-specific spine densities which can be extrapolated to a cumulative number of total spines per neuron (Benavides-Piccione et al., 2013; Eyal et al., 2018). This total number of synapses per neuron is an estimate of the total number of excitatory inputs that an identified postsynaptic neuron may receive (10,000–30,000), which ultimately determines the complexity of input/output transformations. Studies focusing on synapse structure have shown that, in cortical microcircuits, spine shape (Benavides-Piccione et al., 2002; Ofer et al., 2022), spine density (Benavides-Piccione et al., 2002, 2013; Loomba et al., 2022), or total number of spines per neuron can be highly divergent between species. For example, the number of presynaptic vesicles is higher in humans compared to rodents (Yakoubi et al., 2019a,b), and postsynaptic densities are larger (Benavides-Piccione et al., 2002).

Dense (and/or saturated) reconstruction methods using EM have advanced the field of neuroanatomy by revealing not only synapse structure and local connectivity, but also by uncovering the wiring rules of local microcircuits across highly diverse brain regions for rapidly increasing volumes (Helmstaedter et al., 2013; Kasthuri et al., 2015; Motta et al., 2019; Shapson-Coe et al., 2021). However, the *functional* characterization of synaptic transmission remains necessary, to translate static snapshots of wiring principles into dynamic properties of information transfer. These types of experiments are typically performed by dual recordings of synaptically connected neurons (Qi et al., 2020). Synchronous recording of multiple neurons increases the mapping efficiency but is technically demanding (Lefort et al., 2009; Perin et al., 2011; Jiang et al., 2015; Seeman et al., 2018; Peng et al., 2019; Campagnola et al., 2022). This approach also complicates a precise reconstruction of axonal morphology of pre- and postsynaptic neurons due to the extensive overlap of thin axons with relatively large somatodendritic domains. Wiring diagrams have been mapped with particular focus on sensory cortices (Lefort et al., 2009; Cossell et al., 2015; Jiang et al., 2015; Markram et al., 2015; Campagnola et al., 2022). These efforts reveal a complex interplay between morphologically identified cell types, connectivity rates and synapse function (Brown and Hestrin, 2009; Lefort et al., 2009; Campagnola et al., 2022). With the advent of new technologies to define cell types by their transcriptomic profile (Hodge et al., 2019; Scala et al., 2019; Gouwens et al., 2020), it remains crucial to use a consistent and unambiguous procedure to identify neuronal cell types as part of the experimental design. This is particularly important in view of wiring diagrams in which neurons have a variety of postsynaptic partners, and presynaptic neurons tuning their synaptic properties to the cell-type specific identity of the post-synaptic target neuron (Lefort et al., 2009; Peng et al., 2019). In addition, pre-synaptic neurotransmitter release, mean PSP amplitude, short-term depression or facilitation, and recovery from synaptic depression can be highly specific for the connection under study (Campagnola et al., 2022). In short, with only a fraction of all possible cell-to-cell connections characterized in any species, it is too early to generalize synaptic function for highly heterogeneous populations of neuronal cell types.

Fundamental properties of synaptic function include synaptic strength (excitatory/inhibitory postsynaptic potential or EPSP/IPSP, in mV, Figure 1 and Table 1), the dynamics during repetitive stimulation (facilitation/depression, paired pulse ratio), EPSP/IPSP decay kinetics (in ms), ionotropic receptor composition at the postsynaptic membrane, recovery from synaptic depression (τ, in ms) or long-term plasticity (LTP/LTD) rules. We are only beginning to understand how synaptic transmission may vary across species. In this focused review, we aim to summarize recent data on (excitatory) synaptic transmission in rodent and human cortical microcircuits. We compile data on unitary synaptic connections that have been mapped using whole-cell patch clamp electrophysiology. Most of this data comes from synaptic connections between pairs of pyramidal cells in layers 2/3 in which subtypes of pyramidal cells are typically pooled (Figure 1 and Table 1). We also include information on additional cortical layers, and where possible, provide information on specific pre- and postsynaptic cell type (i.e., L4 spiny stellate vs. L4 star pyramid or L6 corticocortical vs. L6 corticothalamic pyramidal cell, Figure 1 and Table 2). Note that in the majority of these connectivity studies, by convention, an extracellular calcium concentration of 2 mM (∼1.7 mM free Ca^2+^) was used; only a small subset used a calcium concentration of 1.3 mM with a minority of studies using concentrations of 1.8 mM, 2.5 mM, or 3.0 mM.

**FIGURE 1:**
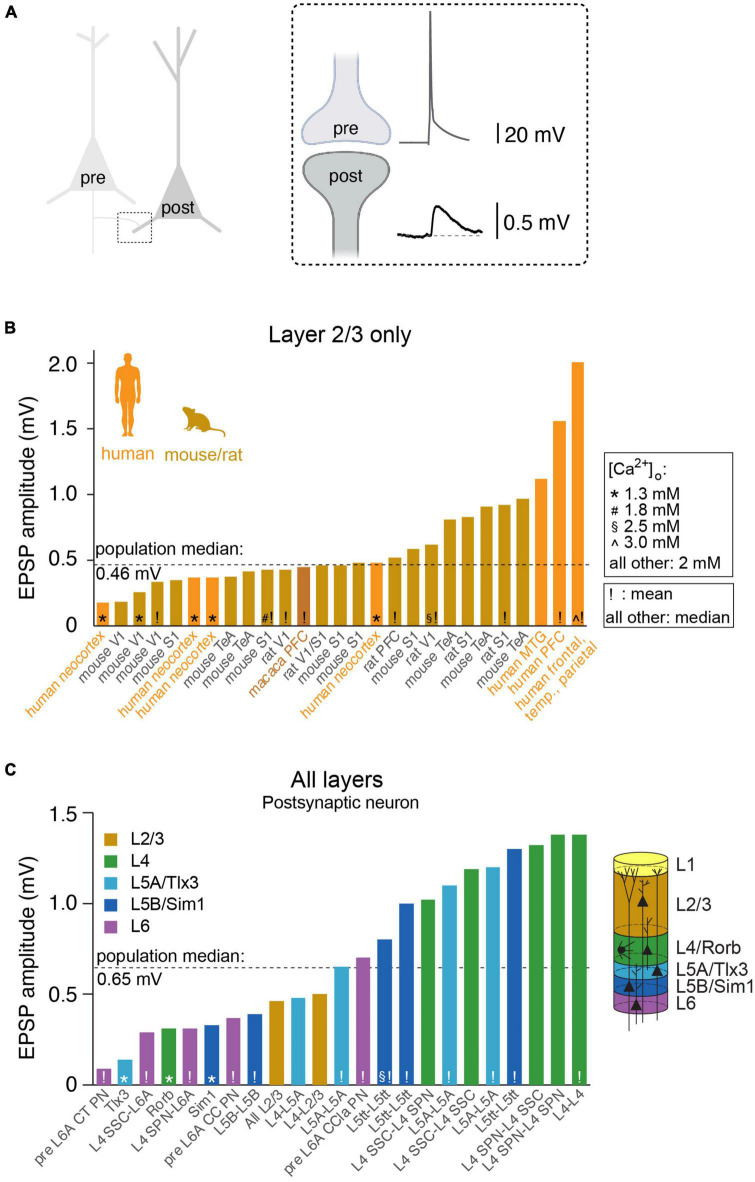
Synaptic strength between pairs of excitatory neurons across species, brain regions, cortical layers and/or cell-types. **(A)** Cartoon illustrating the synaptic connection between the axon of the presynaptic neuron and the dendrite of the postsynaptic neuron. Inset: the action potential in the presynaptic axon triggers neurotransmitter release, evoking a (unitary) excitatory postsynaptic potential (uEPSP) in dendritic spine of the postsynaptic neuron. **(B)** Overview of published uEPSP amplitudes (in mV) for connections exclusively within rodent layer 2/3 or human layer 2 and 3. Symbols indicate external calcium concentration (* 1.3 mM, # 1.8 mM, § 2.5 mM, ∧ 3.0 mM, all other 2.0 mM), exclamation mark indicate that uEPSP amplitude was computed as average (all other: median). Abbreviations: V1: (primary) visual cortex, S1: (primary) somatosensory cortex, TeA: temporal association cortex, PFC: prefrontal cortex, MTG: middle temporal gyrus. **(C)** Analogous to B but for connections between or within additional cortical layers, with particular focus on rodent literature. CT, cortico-thalamic; CC, cortico-cortical; CCla, cortico-claustrum; PN, pyramidal neuron; SPN, star pyramidal neuron; SSC, spiny stellate cell; L5tt, layer 5 thick tufted pyramidal neuron.

**TABLE 1:** uEPSP amplitude (in mV) for excitatory, pyramidal-to-pyramidal connections in cortical layer 2/3 across species.

References	Region	Age	Connection ID	Temp. (°C)	(Ca2+)_o_ (mM)	Stats	Amplitude (mV)
Campagnola et al., 2022	Human neocortex	adult	L3-L2	31–33	1.3	median	0.18
Cossell et al., 2015	Mouse V1	PN22-26	L2/3-L2/3	28	2	median	0.19
Seeman et al., 2018	Mouse V1	PN46.7 ± 6.4	L2/3-L2/3	31–33	1.3	median	0.26
Jiang et al., 2015	Mouse V1	>2 months	L2/3-L2/3		2	average	0.34
Lefort et al., 2009	Mouse S1	PN18-21	L2-L3	35	2	median	0.35
Campagnola et al., 2022	Human neocortex	adult	L2-L2	31–33	1.3	median	0.37
Campagnola et al., 2022	Human neocortex	adult	L3-L3	31–33	1.3	median	0.37
Hunt et al., 2023	Mouse TeA	adult	L2/L3-L2/L3	34	2	median	0.38
Luo et al., 2017	Mouse TeA	PN14-21	L2MN-L2RS	34–35	2	median	0.42
Jouhanneau et al., 2015	Mouse S1	PN22 ± 0.2	L2-L2	37	1.8	average	0.43
Hardingham et al., 2010	Rat V1	PN19-27	L2/3	23-26 or 36	2	average	0.43
Povysheva et al., 2006	macaca PFC	young adult	L2/3-L2/3	31–32	2	average	0.45
Holmgren et al., 2003	Rat V1/S1	PN14-16	L2/3-L2/3	32–34	2	median	0.46
Lefort et al., 2009	Mouse S1	PN18-21	L2-L2	35	2	median	0.46
Lefort et al., 2009	Mouse S1	PN18-21	L3-L3	35	2	median	0.48
Campagnola et al., 2022	Human neocortex	adult	L2-L3	31–33	1.3	median	0.48
Povysheva et al., 2006	Rat PFC	PN19-29	L2/3-L2/3	31–32	2	average	0.52
Lefort et al., 2009	Mouse S1	PN18-21	L3-L2	35	2	median	0.59
Hardingham and Larkman, 1998	Rat visual cortex	PN20-22	L2/3	36	2.5	average	0.62
Luo et al., 2017	Mouse TeA	PN14-21	L2RS-L2MN	34–35	2	median	0.81
Feldmeyer et al., 2006	Rat S1	PN17-23	L2/3-L2/3	34–36	2	median	0.83
Luo et al., 2017	Mouse TeA	PN14-21	pooled	34–35	2	median	0.91
Koester and Johnston, 2005	Rat S1	PN12-16	L2/3	35	2	average	0.92
Luo et al., 2017	Mouse TeA	PN14-21	L2MN-L2MN	34–35	2	median	0.97
Hunt et al., 2023	Human MTG	adult	L2/L3-L2/L3	34	2	median	1.12
Komlosi et al., 2012	Human PFC	male 48 ± 16 years female 53 ± 17 years	L2/L3-L2/L3	36	2	average	1.56
Szegedi et al., 2016	Human frontal, temp., parietal	10–85 years	L2/L3-L2/L3	36–37	3	average	2.01

**TABLE 2:** uEPSP amplitude in mV for excitatory connections between identified cell-types and/or layers in neocortex.

References	Region	Age	Connection ID	Temp. (°C)	[Ca2+]_o_ (mM)	Stats	Amplitude (mV)
Yang et al., 2021b	Rat S1	PN17-21	pre L6A CT PN	30–33	2	average	0.09
Seeman et al., 2018	Mouse V1	PN46.7 ± 6.4	Tlx3	31–33	1.3	median	0.14
Qi and Feldmeyer, 2016	Rat S1	PN18-28	L4 SSC-L6A	32–33	2	average	0.29
Seeman et al., 2018	Mouse V1	PN46.7 ± 6.4	Rorb	31–33	1.3	median	0.31
Qi and Feldmeyer, 2016	Rat S1	PN18-28	L4 SPN-L6A	32–33	2	average	0.31
Seeman et al., 2018	Mouse V1	PN46.7 ± 6.4	Sim1	31–33	1.3	median	0.33
Yang et al., 2021b	Rat S1	PN17-21	pre L6A CC PN	30–33	2	average	0.37
Rollenhagen et al., 2018	Rat S1	PN30-35	L5B-L5B	34–37	2	average	0.39
All L2/3	Mixed	Mixed	All L2/3		Mixed	Mixed	0.46
Feldmeyer et al., 2005	Rat S1	PN17-23	L4-L5A	34-36	2	median	0.48
Feldmeyer et al., 2002	Rat S1	PN17-23	L4-L2/3	34–36	2	median	0.5
Frick et al., 2007	Rat S1	PN24-29	L5A-L5A	33–36	2	average	0.65
Yang et al., 2021b	Rat S1	PN17-21	pre L6A CCla PN	30–33	2	average	0.7
Song et al., 2005	Rat V1	PN12-20	L5tt-L5tt	32–34	2.5	average	0.77
Reyes and Sakmann, 1999	Rat sensorimotor cortex	PN14	L5tt-L5tt	34	2	average	1.0
Feldmeyer et al., 1999	Rat S1	PN12-15	L4 SSC-L4 SPN	36	2	median	1.02
Frick et al., 2007	Rat S1	PN14-16	L5A-L5A	33-36	2	average	1.1
Feldmeyer et al., 1999	Rat S1	PN12-15	L4 SSC-L4 SSC	36	2	median	1.19
Frick et al., 2008	Rat S1	PN18-20	L5A-L5A	32–35	2	average	1.2
Markram et al., 1997	Rat S1	PN14-16	L5tt-L5tt	32–34	2	average	1.3
Feldmeyer et al., 1999	Rat S1	PN12-15	L4 SPN-L4 SSC	36	2	median	1.32
Feldmeyer et al., 1999	Rat S1	PN12-15	L4 SPN-L4 SPN	36	2	median	1.38
Qi et al., 2017	Rat S1	PN17-33	L4-L4	32–33	2	average	1.38

Included studies predominantly quantified synaptic properties for a targeted connection. Extended connection matrices are available in Reyes and Sakmann (1999), Lefort et al. (2009), Jiang et al. (2015), and Campagnola et al. (2022).

A recent study has shown that the total extracellular Ca^2+^ in human cerebral spinal fluid can be as low as 1.2 mM (∼1.0 mM free Ca^2+^ (Forsberg et al., 2019). Since the Ca^2+^ concentration has been shown to affect neuronal excitability, presynaptic release probability and short-term synaptic plasticity (Molnar et al., 2016; Forsberg et al., 2019), it is crucial to incorporate this parameter into the comparison of synaptic strength across different studies. Recording temperature also affects synaptic release probability (hence: uEPSP amplitude), failure rate, reliability (Hardingham and Larkman, 1998), or synaptic plasticity (Klyachko and Stevens, 2006) and the overview thus provides both Ca^2+^ concentration and recording temperature at which the synaptic strength was quantified.

Perhaps the best-studied neuronal microcircuits are the primary somatosensory (S1) and primary visual (V1) cortices (Reyes and Sakmann, 1999; Lefort et al., 2009; Feldmeyer et al., 2013; Jiang et al., 2015; Markram et al., 2015; Seeman et al., 2018; Campagnola et al., 2022). Depending on the specific connection between excitatory cell types within or across layers, the EPSP amplitude at the soma can vary considerably (Figure 1 and Table 2). For example, the EPSP amplitude for the unitary connection between presynaptic L6A cortico-thalamic pyramidal neurons and a L6A excitatory cell type as postsynaptic target (in S1) is 0.09 mV (Yang et al., 2021b). A connection with a much larger mean unitary EPSP (uEPSP) amplitude is between L4 excitatory neurons (i.e., 1.38 mV) (Qi et al., 2017) and multiple EPSP amplitude values have been reported to fall within this range (Figure 1 and Tables 1, 2). The unitary synaptic connection between pyramidal neurons in cortical layers 2 and 3 has been characterized in several studies. They show substantial differences in the mean or median uEPSP amplitude for L2/3 pyramidal-to-pyramidal cell connections across different cortical regions (PFC, TeA, S1, V1, MTG) and species (mouse, rat, macaque, human; total range: 0.18–2.01 mV, Figure 1 and Table 1). Experimental or analytical conditions such as developmental stage (i.e., juvenile, adolescent, adult), external Ca^2+^ (1.3, 1.8, 2.0, 2.5 or 3.0 mM), temperature or reported mean/median could certainly influence the reported uEPSP amplitude, but it is also likely that the synaptic strength is dependent on the connection established between different L2/3 pyramidal cell types (Deitcher et al., 2017; Hodge et al., 2019; Berg et al., 2021; Hunt et al., 2023). Pyramidal-to-interneuron or interneuron-to-pyramidal cell connections are typically stronger (Molnar et al., 2016; Wilbers et al., 2023), but here we focus on synaptic transmission between pairs of excitatory neurons. In this context, it is important to emphasize that amplitude distributions are typically skewed with the majority of connections showing small amplitudes and a long tail of stronger connections (Feldmeyer et al., 1999; Holmgren et al., 2003; Song et al., 2005; Cossell et al., 2015; Seeman et al., 2018; Hunt et al., 2023). We would therefore argue that the median (and 1st-3rd interquartile ranges) should be consistently reported as it is more representative for the skewed population data. Ideally, these population statistics are also supplemented with the full range (min/max) of the population data as the extremes of the lognormal distribution may have biologically relevant functions (Szegedi et al., 2016, 2017). Synaptic connections with small mean uEPSP amplitudes may also fall below the detection power [Seeman et al., 2018; Supplementary Figure 14 in Campagnola et al. (2022) and Supplementary Figure 3 in Qi and Feldmeyer (2016)], especially when using a limited number of sweeps to probe for the presence of a connection. The combination of small amplitude connections and low detection power increases the false negative rate, underestimates the true connectivity, and may lead to an overestimation of mean EPSP amplitude of a given synaptic connection type. It is therefore perhaps not surprising that connections with a small uEPSP amplitude may be missed by electrophysiological recordings from the soma [but see Yang et al. (2021b)], but are reliably detected using EM techniques (Loomba et al., 2022). However, the use of EM techniques has also limitations when studying wiring diagrams because the axonal arbors occupy much larger volumes relative to the tissue blocks currently processed for EM examination (Oberlaender et al., 2011; Narayanan et al., 2015; Winnubst et al., 2019). Therefore, cross-scale techniques including correlated anatomical and physiological measurements are still urgently needed to generate a comprehensive, functional wiring diagram for microcircuits of interest.

For a given connection type, population distributions of uEPSP amplitude can thus show a pronounced skew with a subset of unitary connections being particularly strong and even sufficiently large to evoke AP firing (Feldmeyer et al., 1999, 2006; Holmgren et al., 2003; Silver et al., 2003; Song et al., 2005; Frick et al., 2008; Lefort et al., 2009). These exceptionally strong excitatory connections are attractive to study because *in vivo* recordings from primary somatosensory and primary visual cortices have shown that a small subset of excitatory neurons show a particularly strong response to sensory stimulation, while the majority of neurons respond with only a subthreshold depolarization or not all, [e.g., (Brecht et al., 2003; de Kock et al., 2007; Kerr et al., 2007; O’Connor et al., 2010; Crochet et al., 2011; Barth and Poulet, 2012; Cossell et al., 2015; Barz et al., 2021)]; these neurons can be referred to as ‘high responders’. A systematic analysis of the properties of these ‘high responder’ neurons and the impact of large uEPSPs is still lacking and as such a matter of debate. It would be interesting to determine whether particularly strong intracortical synapses go hand in hand with reliable sensory representation *in vivo* (Yassin et al., 2010).

For neurons with dendrites that are electrically relatively compact [e.g., rodent neurons, (Beaulieu-Laroche et al., 2018)], detection power may not be a major issue as distal synapses can still have a profound impact on the somatic membrane potential. However, cortical neurons of human brain have much longer dendrites as well as increased branching (Mohan et al., 2015). The outcome of these extended morphologies is a huge capacitance load on electrical signals traveling from distal regions to the soma resulting in electrically isolated subcompartments (Eyal et al., 2014; Beaulieu-Laroche et al., 2018; Gidon et al., 2020). Therefore, the risk of false negative connections increases substantially when probing human cortical circuits (Seeman et al., 2018). The increased path length from dendrite to soma and potentially increased dendritic attenuation opens the possibility of evolutionary adaptation of human neurons. For example, one neurophysiological property that may compensate for this, is active dendritic electrogenesis, which has been extensively documented for somatosensory L5 thick tufted neurons (Larkum et al., 1999, 2022) and to some extent in pyramidal cells in layer 6 and superficial cortical layers (Ledergerber and Larkum, 2010, 2012). Depending on the type of synaptic input, dendritic electrogenesis will initiate voltage-dependent Ca^2+^ spikes in the apical dendrites or NMDA spikes in the basal dendrites and the apical tuft dendrites; these serve to amplify synaptic signals and ensure proper propagation to the soma (Larkum et al., 2022). Alternatively, it was suggested that human L2/L3 neurons may have a lower membrane capacitance compared to rodents (human: 0.5 μF/cm^2^, rodent: 1.0 μF/cm^2^, [(Eyal et al., 2016), but see Gooch et al. (2022)]. This potential adaptation could be specific to L2/L3 neurons (Beaulieu-Laroche et al., 2018) and translates to enhanced synaptic charge-transfer from dendrites to soma and ultimately similar EPSP amplitudes across species (Figure 1 and Table 1). Given the extended morphologies and increased path length of human neurons, it is even more important to understand the active properties of human dendrites in order to understand the passive and active propagation of synaptic inputs and ultimately neuronal input/output transformations (Gidon et al., 2020; Beaulieu-Laroche et al., 2021; Kalmbach et al., 2021; Testa-Silva et al., 2022).

Synaptic connections (and thus uEPSP amplitudes) are also affected by neuromodulatory transmitters such as acetylcholine and monoamines, by neuropeptides, and by many hormones all of which act by activating or deactivating ionotropic ion channels and/or G protein-coupled receptors (GPCRs). The release of these neuromodulators is dependent on circadian rhythm, behavioral state and age. Examples of neuromodulator receptors that cause an increase or decrease in the synaptic release probability include muscarinic and nicotinic acetylcholine receptors, as well as several types of serotonin and adenosine receptors [for reviews see Puig and Gulledge (2011), Radnikow and Feldmeyer (2018), Yang et al. (2021a)]. In addition, the released neurotransmitter may diffuse out of the synaptic cleft (‘spill over’) and affect the synaptic release probability by acting on G protein-coupled receptors (such as metabotropic glutamate receptors or GABA_*B*_ receptors) in the perisynaptic membrane of the same or neighbouring presynaptic terminals (Kullmann and Asztely, 1998; Uchida et al., 2012; Wild et al., 2015). These neuromodulators are present in the cerebrospinal fluid at low micromolar concentrations and therefore affect synaptic release even in the absence of pharmacological intervention. The discovery of divergent gene expression of a major neuromodulatory receptor system between human and mouse (i.e., serotonin, (Hodge et al., 2019), calls for a detailed characterization of the interplay between baseline synaptic transmission in humans and the impact of the major neuromodulator systems.

**Synaptic connections in the human neocortex**

To date, only a small number of studies exist that have examined synaptic transmission at autapses (Yin et al., 2018) or between pairs of excitatory neurons in human brain (Komlosi et al., 2012; Szegedi et al., 2016; Seeman et al., 2018; Peng et al., 2019; Campagnola et al., 2022; Hunt et al., 2023). The availability of human brain tissue is typically a by-product of neurosurgical resection of epileptic foci or tumors. This provides a window of opportunity to study synaptic transmission and quantify how neurons with overall larger morphologies, extended dendrites and most likely many more synaptic inputs than rodents deal with synaptic inputs. With only a handful of studies available, it is too premature to generalize, but the emerging data show common and divergent properties of synaptic transmission across species.

Measured at the soma, uEPSP amplitude for pyramidal-to-pyramidal cell connections in layer 2/3 across species is remarkably consistent (median ALL 0.46 mV, mouse: 0.43 mV, rat: 0.57 mV, macaque: 0.45 mV, human: 0.48 mV, Figure 1 and Table 1). This suggests that obvious differences in the principles of brain organization or neuronal architecture do not affect the fundamental unit of synaptic information transfer. Examples of these differences in organizational principles across species include brain mass [mouse 0.417 g, human 1,508 g, (Herculano-Houzel, 2009), the number of cortical neurons (mouse: 2 billion, human: 16 billion, (Herculano-Houzel, 2009), estimated spine count on an individual L2/3 pyramidal neuron (mouse: 10,000, human: 30,000, (Eyal et al., 2018) or total dendritic length (mouse: 5.3 mm, human: 14.5 mm, (Mohan et al., 2015)]. These are just a few example differences, but can easily be extended to dendritic path length, synapse density, and many more (Benavides-Piccione et al., 2002; Yakoubi et al., 2019a,b; Loomba et al., 2022). This certainly does not mean that everything is equal. A consistent finding now reported across two independent sites is that excitatory transmission is stronger in L2/L3 pyramidal-to-pyramidal connections for human compared to L2/3 in mouse (Campagnola et al., 2022; Hunt et al., 2023), which could be (in part) explained by increased contribution of NMDA receptor activation during unitary synaptic transmission in humans but not mice. Alternatively, increased synaptic strength can be the outcome of a difference in presynaptic release probability (Hunt et al., 2023). A second consistent finding is that recovery from synaptic depression is faster in humans compared to mice (Testa-Silva et al., 2014; Campagnola et al., 2022; Hunt et al., 2023). These differences have implications for the cellular information transfer, signal flow within neuronal microcircuits and thus ultimately cognition and mental ability (Goriounova et al., 2018).

## Conclusion and outlook

The synapse is the fundamental building block of neuronal microcircuits across species. The anatomical features (Benavides-Piccione et al., 2002), functional properties (Seeman et al., 2018; Campagnola et al., 2022; Hunt et al., 2023), and plasticity dynamics (Testa-Silva et al., 2014) differ when comparing pyramidal-to-pyramidal cell connections in cortical L2/3 in human and mouse. Comparable differences in anatomy (Yakoubi et al., 2019a,b) or physiology (Molnar et al., 2016) have also been described in deeper layers or pyramidal-to-interneuron connections, respectively, suggesting a spectrum of human-specific adaptations in synaptic transmission. As we are only beginning to see the tip of the iceberg and much remains unknown about synaptic transmission in human (and non-human primates), it will be critical to continue efforts to study synaptic transmission in the human brain between identified cell types. This will accelerate efforts to build realistic biophysical models of individual neurons (Eyal et al., 2018; Hunt et al., 2023), microcircuit simulations (Markram et al., 2015; Joglekar et al., 2018) and ultimately facilitate a comprehensive understanding of human cognitive abilities in health and disease.

## Author contributions

CK: Conceptualization, Funding acquisition, Writing—original draft, Writing—review and editing. DF: Conceptualization, Funding acquisition, Writing—original draft, Writing–review and editing.
